# Intestinal Lesions Are Associated with Altered Intestinal Microbiome and Are More Frequent in Children and Young Adults with Cystic Fibrosis and Cirrhosis

**DOI:** 10.1371/journal.pone.0116967

**Published:** 2015-02-06

**Authors:** Thomas Flass, Suhong Tong, Daniel N. Frank, Brandie D. Wagner, Charles E. Robertson, Cassandra Vogel Kotter, Ronald J. Sokol, Edith Zemanick, Frank Accurso, Edward J. Hoffenberg, Michael R. Narkewicz

**Affiliations:** 1 Section of Pediatric Gastroenterology, Hepatology and Nutrition, Department of Pediatrics, University of Colorado School of Medicine and Children’s Hospital Colorado, Aurora, CO, United States of America; 2 Section of Pediatric Pulmonary Medicine, Department of Pediatrics, University of Colorado School of Medicine and Children’s Hospital Colorado, Aurora, CO, United States of America; 3 Department of Pediatrics, University of Colorado School of Medicine and Children’s Hospital, Aurora, Colorado, United States of America; 4 Colorado Clinical and Translational Sciences Institute, University of Colorado School of Medicine, Aurora, CO, United States of America; 5 Department of Infectious Diseases, University of Colorado Denver, Aurora, Colorado, United States of America; 6 Department of Biostatistics and Informatics, Colorado School of Public Health, University of Colorado Denver, Aurora, CO, United States of America; 7 Department of Molecular, Cellular, and Developmental Biology, University of Colorado Boulder, Boulder, Colorado, United States of America; University Medical Center Utrecht, NETHERLANDS

## Abstract

**Background and Aims:**

Cirrhosis (CIR) occurs in 5–7% of cystic fibrosis (CF) patients. We hypothesized that alterations in intestinal function in CF contribute to the development of CIR. Aims: Determine the frequency of macroscopic intestinal lesions, intestinal inflammation, intestinal permeability and characterize fecal microbiome in CF CIR subjects and CF subjects with no liver disease (CFnoLIV).

**Methods:**

11 subjects with CFCIR (6 M, 12.8 yrs ± 3.8) and 19 matched with CFnoLIV (10 M, 12.6 yrs ± 3.4) underwent small bowel capsule endoscopy, intestinal permeability testing by urinary lactulose: mannitol excretion ratio, fecal calprotectin determination and fecal microbiome characterization.

**Results:**

CFCIR and CFnoLIV did not differ in key demographics or CF complications. CFCIR had higher GGT (59±51 U/L vs 17±4 p = 0.02) and lower platelet count (187±126 vs 283±60 p = 0.04) and weight (-0.86 ± 1.0 vs 0.30 ± 0.9 p = 0.002) z scores. CFCIR had more severe intestinal mucosal lesions on capsule endoscopy (score ≥4, 4/11 vs 0/19 p = 0.01). Fecal calprotectin was similar between CFCIR and CFnoLIV (166 μg/g ±175 vs 136 ± 193 p = 0.58, nl <120). Lactulose:mannitol ratio was elevated in 27/28 subjects and was slightly lower in CFCIR vs CFnoLIV (0.08±0.02 vs 0.11±0.05, p = 0.04, nl ≤0.03). Small bowel transit time was longer in CFCIR vs CFnoLIV (195±42 min vs 167±68 p<0.001, nl 274 ± 41). *Bacteroides* were decreased in relative abundance in CFCIR and were associated with lower capsule endoscopy score whereas *Clostridium* were more abundant in CFCIR and associated with higher capsule endoscopy score.

**Conclusions:**

CFCIR is associated with increased intestinal mucosal lesions, slower small bowel transit time and alterations in fecal microbiome. Abnormal intestinal permeability and elevated fecal calprotectin are common in all CF subjects. Disturbances in intestinal function in CF combined with changes in the microbiome may contribute to the development of hepatic fibrosis and intestinal lesions.

## Introduction

Cystic Fibrosis (CF) is the most common lethal genetic disease in North America, with about 30,000 affected individuals, with approximately 1,000 new cases diagnosed yearly. Although pulmonary disease is the most common cause of mortality [1–3], liver disease is the third leading cause of death, accounting for 2.5% of overall mortality [4,5]. Autopsy data have demonstrated up to 72% of adults with CF have some form of liver involvement [6]. However, advanced liver disease, defined as multilobular cirrhosis frequently with portal hypertension, occurs in only 5–10% of individuals with CF [4,5,7]. Although most patients likely have some degree of liver involvement because cystic fibrosis transmembrane regulatory protein (CFTR), the causative gene, is expressed in bile duct epithelia, the pathogenesis of advanced liver disease in CF is still largely speculative. Cirrhosis occurs predominantly in individuals with pancreatic insufficiency and severe mutations in the *CFTR* gene, however no CFTR genotype/hepatic phenotype correlation has been identified. A recent investigation of genetic factors that may predispose to cirrhosis in CF identified the PIZ heterozygote state for alpha-1 antitrypsin (*SERPINA1*) to be associated with an odds ratio of 5 and population attributable risk of 7% for the development of cirrhosis, however, this was present in only 9% of the subjects with cirrhosis[8]. Other factors inconsistently associated with cirrhosis in CF have been male sex, meconium ileus, and TGF-β1 polymorphisms [7–10].

The current model of the pathogenesis of CF liver disease suggests that inspissated bile from deficient CFTR function in biliary epithelial cells leads to obstruction of intrahepatic bile ducts, accumulation of toxic bile acids, depletion of hepatic antioxidants, subsequent liver cell injury and inflammation, and activation of hepatic stellate cells which generate fibrosis and eventual cirrhosis [11]. We propose an alternative theory for the development of CF cirrhosis, involving the gut-liver axis in which intestinal mucosal inflammation and ulceration, bacterial overgrowth of pathogenic microbiota and increased permeability of the small intestine promote translocation of bacterial factors into the portal circulation that activate hepatic inflammation and pathways that generate fibrogenesis with the subsequent development of increased portal fibrosis. These pathways have been demonstrated to be involved in the pathogenesis of a number of other liver diseases including cholestatic injury and steatohepatitis, both of which are present in CF liver disease [12–14] [15,16] [17].

Data that support this proposed model of CF cirrhosis are the following. Studies in the *cftr* knockout mouse have shown that intestinal inflammation is associated with the development of liver disease[18,19]. A recent study demonstrated that greater than 70% of pancreatic insufficient CF patients had visible intestinal inflammatory lesions on capsule endoscopy [20]. Elevated fecal calprotectin levels, suggestive of intestinal inflammation, have been reported in CF patients [20,21] as well as adults with cirrhosis not due to CF [22]. Furthermore, there is increased intestinal permeability in CF patients [21,23–28] and in individuals with non-CF cirrhosis. This increased permeability has been reported prior to development of cirrhosis or portal hypertension in other hepatic conditions [29–31]. The etiology of intestinal mucosal inflammation and increased intestinal permeability in CF is unknown, but alterations in local bacterial species in the small bowel are suspected to play a role. These potential mechanisms have not been investigated in CF liver disease. The purpose of this study was to conduct a pilot study to determine the frequency of intestinal lesions and inflammation, alterations in intestinal permeability and characterization of the fecal microbiome in patients with CF with and without cirrhosis as an initial investigation of the potential role of the gut-liver axis in CF liver disease.

## Methods

All aspects of this study were reviewed and approved by the Colorado Multiple Institutional Review Board and informed consent was signed by subjects 18 years and older or parents/guardians for younger subjects; assent was given by all subjects age 7–17 years Protocol Number: 10–1404.

### Study Subjects

Subjects were recruited from our local CF clinic population in Colorado. Written informed consent was obtained from subjects 18 years and older or parents/guardians for younger subjects; assent was given by all subjects age 7–17 years. All co-authors had access to the study data and reviewed and approved the final manuscript

This was a prospective case-controlled study. There were two groups of subjects: pancreatic insufficient CF subjects with cirrhosis (CFCIR) and pancreatic insufficient CF subjects with no clinical or laboratory evidence of liver disease (CFnoLIV) (matched controls). Inclusion criteria were:

1. A diagnosis of CF confirmed by a sweat chloride > 60 mEq/L or the presence of 2 disease causing CFTR mutations with end organ involvement.
2. Age 7–35 years.
3. Presence of pancreatic insufficiency as defined by any one of the following: fecal elastase <100 mg/gm, a 72-hour fecal fat with coefficient of absorption < 85%, presence of two copies of CFTR mutations associated with pancreatic insufficiency, or clinical evidence of pancreatic insufficiency requiring pancreatic enzyme replacement therapy.
4. CFCIR group was defined by at least one of the following: a) clinical evidence of portal hypertension (splenomegaly, ascites, esophageal or gastric varices), b) CT, MRI or ultrasound findings consistent with cirrhosis, or c) liver biopsy histology with stage 5 or 6 Ishak score for fibrosis.
5. CFnoLIV group was defined by no clinical, radiographic or biochemical evidence or history of liver disease or portal hypertension and a normal serum ALT and GGT in the past year.

The CFnoLIV subjects were matched 2:1 to CFCIR by age (±2 years for those <19 years of age and ±5 years for those ≥19 years of age) and *Pseudomonas* status as indicated by any positive culture in the last 12 months.

Subjects were excluded if they had pancreatic sufficiency, chronic liver disease due to a cause other than CF, solid organ or bone marrow transplantation, known inflammatory bowel disease or celiac disease, daily use of NSAID’s in the previous 2 weeks, intestinal surgery in the past year, presence of a known intestinal stricture, inability to swallow the video capsule safely or were pregnant. Subjects with a history of meconium ileus during infancy, intestinal surgery more than one year prior to the study, or a history of distal intestinal obstruction syndrome were further screened for intestinal patency using the PillCam patency capsule (Given Imaging) as previously described [20]. Any retention of this capsule in the small bowel after 30 hours resulted in elimination from the study. (6 patients screened, 0 eliminated).

### Study Design

Following informed consent and enrollment, subjects were admitted to the Clinical Translational Research Center (CTRC) at Children’s Hospital Colorado and clinical and demographic data were obtained from an interview and review of the medical record. The following studies were conducted on the following day: video capsule endoscopy, intestinal permeability testing and stool collection.

### Video capsule endoscopy

All participants received a bowel preparation consisting of a clear liquid diet with 3 to 4 doses (17gm per dose) of Miralax dissolved in 8 ounces clear liquid the afternoon prior to video capsule administration. The following morning, participants consumed a single liquid dose of 200mg erythromycin (to enhance gastric emptying) and 120mg simethicone (to reduce gas that may interfere with the video images) 30–60 minutes prior to capsule administration. Participants then swallowed the video capsule (Given Imaging, Yoqneam, Israel) and images were recorded for the following 8 hours. This capsule takes 156° degree images which are transmitted to a wireless receiver located in a harness worn by the subject during the study. The subjects were instructed to not eat or drink for 120 minutes following the swallowing of the capsule and then to eat a light meal 4 hours into the study. Following completion of the image acquisition, the video endoscopic images were uploaded into a dedicated PC with RAPID image software (Given Imaging) for later analysis. De-identified video capsule images were independently analyzed by two reviewers blinded to the subject group (TF and EH), and intestinal findings were scored by the system of Maiden et al. [32], and recently utilized by Werlin at al. in CF patients [20]. In this system, 1 point is assigned for each individual presence of petechiae, red spots, erythema, reddened folds, denuded areas and erosions/ulcers as indicators of intestinal inflammation. A priori we assigned a score of ≥4 as an indication of more significant intestinal lesions to compare more severe to less severe macroscopic involvement based on the distribution of scores in the previous studies. We also evaluated, but did not include in the score, the presence of blood. A priori we assigned a score of ≥4 as an indication of more significant intestinal lesions to compare more severe to less severe macroscopic involvement. Gastric emptying and small bowel transit times were calculated by each reviewer based on the time to the first gastric, duodenal and cecal images.

### Intestinal Permeability Testing

Intestinal permeability was assessed using the lactulose/mannitol (Lac/Man) urinary excretion ratio following the protocol described by van Elburg et al [33]. Two hours after video capsule ingestion, the subject simultaneously consumed 5 gm of lactulose (large sized sugar) and 2 gm of mannitol (medium sized sugar) dissolved in water to a total volume of 100ml. The subsequent first urine was discarded. Thereafter urine was collected for 5 hours in an opaque plastic container with 0.5 ml of 20% chlorhexidine digluconate added as preservative. At 5 hours, the total volume of the sample was measured, and a 30 ml aliquot of collected urine was frozen at -70° until analysis [34]. The concentrations of lactulose and mannitol in the urine were determined by HPLC using [35] and the Lac/Man ratio was expressed as the percentage of lactulose excreted over the percentage of mannitol excreted. The ratio of lactulose to mannitol was then calculated as an estimate of intestinal permeability. Normal values have been previously determined as Lac/Man ≤ 0.03 [29]. A ratio >0.03 reflects increased small intestinal permeability, with higher ratios indicating greater permeability.

### Fecal calprotectin

The first stool produced after admission to the CTRC was collected and frozen at -70° C for calprotectin analysis. Calprotectin was extracted from 80–120 mg of stool and determined by quantitative ELISA assay using the PhiCal ELISA kit (Genova diagnostics, Asheville, NC) following the manufacturer’s instructions [36]. Calprotectin levels were reported as mcg/g stool, with the normal ≤50, borderline >50–120 and abnormal >120 mcg/g of stool. This test has been shown to correlate with the severity of intestinal inflammation in inflammatory bowel disease patients, and has been shown to be accurate and reproducible[37–42].

### Fecal Microbiome Analysis

Bacterial profiles were analyzed by broad-range PCR of 16S rRNA genes and phylogenetic sequence analysis. DNA was extracted using the UltraClean fecal DNA kit (MoBio, Inc). Amplicons of the 16S rRNA gene were generated via broad-range PCR (30 cycles) using barcoded primers[43] modified with sequences required for Illumina sequencing-by-synthesis (~250 b.p. V4 region; primers 538F and 806R [44]). PCR yields were normalized using a SequalPrepTM kit (Invitrogen, Carlsbad, CA), pooled, lyophilized, and gel purified, as previously described [45,46]. Illumina paired-end sequencing was performed by Dr. Frank’s laboratory using an Illumina MiSeq personal sequencing platform and the 500-cycle MiSeq Reagent Kit version 2.

Paired-end sequences were sorted by sample via barcodes and assembled using phrap[47–49]. Assembled sequence ends were trimmed over a moving window of 5 nucleotides until average quality met or exceeded 20. Trimmed sequences with more than 2 ambiguities or shorter than 200 nt were discarded. Potential chimeras, identified with Uchime (usearch6.0.203_i86linux32) [50] using the Schloss Silva reference sequences[51], were removed from subsequent analyses. The remaining sequences were aligned and classified with SINA (1.2.11)[52] using the 244,077 bacterial sequences in Silva 111NR[53] as reference configured to yield the Silva taxonomy. This process produced a total of 1,912,465 high-quality 16S rRNA sequences, with a median of 32,163 sequences (IQR 14,193–66,328) per subject. All 16S amplicon libraries were sequenced to >99% coverage, as measured by Good’s coverage index. Raw sequence reads were deposited in the NCBI short read archive under BioProject ID PRJNA257183.

### Statistical Analysis

Demographic and baseline clinical characteristics were summarized using descriptive statistics by group. Mean and standard deviation (or median and inter-quartile as appropriate) were calculated for continuous variables and count and percent were used for summarizing categorical variables. Capsule endoscopy scores of intestinal inflammation were categorized by Maiden’s classification [20,32]. A cut-off of 4 in Maiden’s score was applied to dichotomize the classification as severe intestinal lesions (≥4) or not. A Chi-Square test was used to compare the lesions between CFCIR and CFnoLIV controls. Correlations between the number of lesions (primary measure) and each of the secondary measures (calprotectin, percent lactulose or percent mannitol absorbed and Lac/Man ratio) were evaluated within groups using Spearman Rank Correlation test. Student’s T test was used to test for differences between CFCIR and CFnoLIV for fecal calprotectin and Lac/Man results. A cutoff of 120 mcg/gm of stool was used for the calprotectin data to dichotomize into normal (<120)/not normal. Stratified conditional logistic regression was employed to test whether the proportion of abnormal calprotectin was higher in CFCIR than CFnoLIV subjects. A p-value of < 0.05 was used for statistical significance.

Ecological indices [54] of richness (e.g., S_obs_, S_chao_), diversity (e.g., Shannon’s diversity [H_o_] and evenness [H_o_/H_max_]), and coverage (e.g., Good’s index) were calculated with the software package Explicet.[55] These indices were estimated through bootstrap resampling (1000 replicates) of the OTU distributions obtained from each specimen. Relative abundance was calculated by dividing sequence counts by the total number of sequences obtained for a sample. For consistency with the analysis of the clinical variable, stratified logistic regression was used to evaluate each bacterial taxa across CFCIR and CFnoLIV groups. The stratification accounts for the subject matching. Relative abundance measures were compared across the dichotomous clinical variables (Capsule score, red spots, varices, etc) using a Wilcoxon rank based test. Analyses were performed at the phylum, family and genus level. Because of the pilot nature of this study, we did not correct p-values for multiple comparisons. Rather, we interpret the p-values as indicating candidate taxa for follow-up testing.

## Results

Eleven CFCIR and 19 CFnoLIV subjects were enrolled in and completed this study. There were no significant differences in age, gender, pulmonary function, CF related diabetes, frequency of use of chronic azithromycin, recent hospitalizations or BMI between the two groups (Table 1). The CFCIR subjects had lower height and weight-for-age z scores. More subjects with CFCIR had undergone intestinal surgery and there was a trend towards more individuals with CFCIR with a history of meconium ileus. As expected, subjects with CFCIR had higher ALT and GGTP, lower platelet counts and evidence of portal hypertension compared to CFnoLIV (Table 1). More subjects with CFnoCIR were receiving inhaled antibiotics (Table 1).

**Table 1:** Patient Demographics and Clinical Characteristics.

	CFnoLIV (n = 19)	CFCIR (n = 11)	*p* value
Age (mean ± SD, years)	12.8 ± 3.8	12.6 ± 3.4	0.83
Male Gender (n, % males)	10 (52.6%)	6 (54.6%)	0.92
Weight for age z score (mean ± SD)	0.30 ± 0.9	-0.86 ± 1.0	0.002
Height for age z score (mean ± SD)	0.15 ± 0.7	-0.67 ± 0.8	0.006
BMI for age z score (mean ± SD)	-0.003 ± 0.7	-0.28 ± 0.9	0.36
FEV1%Predicted	82.3 ± 7.6	79.7 ± 11.4	0.46
ΔF508 homozygous n (%)	11 (57.9%)	8 (72.7%)	0.47
CF Related Diabetes n (%)	3 (15.8%)	1 (9.1%)	1.00
Meconium Ileus n (%)	2 (10.5%)	5 (45.5%)	0.07
Intestinal surgery n (%)	1 (5.3%)	4 (36.4%)	0.047
Chronic Pseudomonas n (%)	6 (31.6%)	2 (20%)	0.06
Hospitalized past Year n (%)	7 (36.8%)	3 (27.3%)	0.70
Oral Azithromycin n (%)	10 (52.6%)	5 (45.5%)	0.70
Inhaled Antibiotics n (%)	1 (5.3%)	4 (40%)*	0.02
Evidence of portal hypertension	0	8 (72%)	<0.001
Selected laboratory parameters, mean ± SD			
ALT (IU/L)	24 ± 10	46 ± 33	0.03
GGT (IU/L)	17 ± 4	59 ± 51	0.018
Hemoglobin (gm/dL)	14.2 ± 1.2	13.7 ± 1.6	0.25
Platelet count (1000/μl)	283 ± 60	187 ± 126	0.037
Albumin (gm/dl)	4.3 ± 4.2	4.0 ± 0.5	0.20

CFnoLIV: CF subjects with no evidence of liver disease (normal exam and ALT), CFCIR: CF subjects with cirrhosis.

*1 subject had missing value

**Fig 1:**
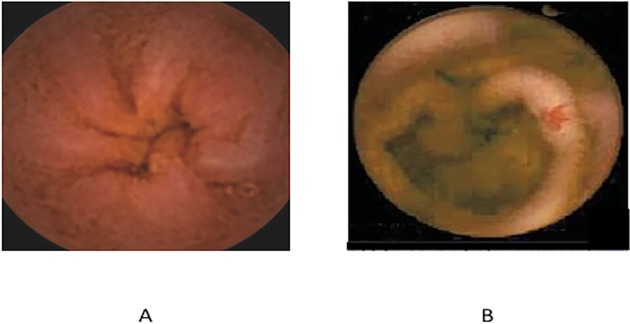
Video capsule endoscopy results. Representative findings from a normal video capsule endoscopy image (A) and an abnormal video capsule endoscopy demonstrating a red spot (B).

The findings for video capsule endoscopy are shown in Table 2. The individual measurements were not different between groups; however more CFCIR subjects had advanced lesions with a score of 4 or greater. Representative lesions seen on capsule endoscopy are presented in Fig. 1. There were no differences in gastric emptying time, but the small bowel transit time was significantly slower in CFCIR compared to CFnoLIV (195±42 vs. 167±68 minutes, p<0.001, Table 3).

**Table 2:** Video Capsule Endoscopy Scores.

	Petechiae	Erythema	Denuded Areas	Ulcerations	Blood	Total Score> = 4
CFnoLIV n = 19Count (%)	10 (53%)	0 (0%)	0 (0%)	3 (16%)	4 (21%)	0 (0%)
CFCIR n = 11, Count (%)	4 (37%)	0 (0%)	1 (9%)	4 (37%)	(27%)	4 (37%)

Scores derived from the scoring system of Maiden et al [32], where 1 point is assigned for each individual presence of each finding (except blood).

CFnoLIV: CF subjects with no evidence of liver disease (normal exam and ALT), CFCIR: CF subjects with cirrhosis.

**Table 3:** Gastric emptying and intestinal transit times and permeability results.

	CFnoLIV	CFCIR	p value
Gastric empting (minutes)*	27 ± 19	49 ± 50	0.43
Small Bowel Transit (minutes)	167 ± 68	195 ± 42	<.001
Percent lactulose absorbed	3.8 ± 2.5	2.7 ± 1.3	0.20
Percent mannitol absorbed	34.4 ± 13.2	32.2 ± 9.2	0.68
lactulose absorbed/mannitol absorbed	0.11 ± 0.05	0.08 ± 0.02	0.042
fecal calprotectin (mcg/gm stool)	137 ± 188	167 ± 175	0.58

*Values are mean ± SD.

CFnoLIV: CF subjects with no evidence of liver disease (normal exam and ALT), CFCIR: CF subjects with cirrhosis.

Intestinal permeability measured by the Lac/Man ratio was abnormal (>0.03) in 27/28 of the subjects in whom data was available. However, there was a slightly higher Lac/Man ratio in CFnoLIV compared to CFCIR (0.11±0.05 vs. 0.08±0.02, p = 0.04). Neither the percent absorption of lactulose nor mannitol was different between the two groups (Table 3).

Mean fecal calprotectin values were not significantly different between CFCIR and CFnoLIV (CFCIR 167 μg/g ±175 vs CFnoLIV 137 ± 193 p = 0.58). There was no difference between the number of CFCIR and CFnoLIV subjects who had an abnormal fecal calprotectin of >120 mcg/gm (5/11, 45% vs 5/19 26% p = 0.43).

**Fig 2:**
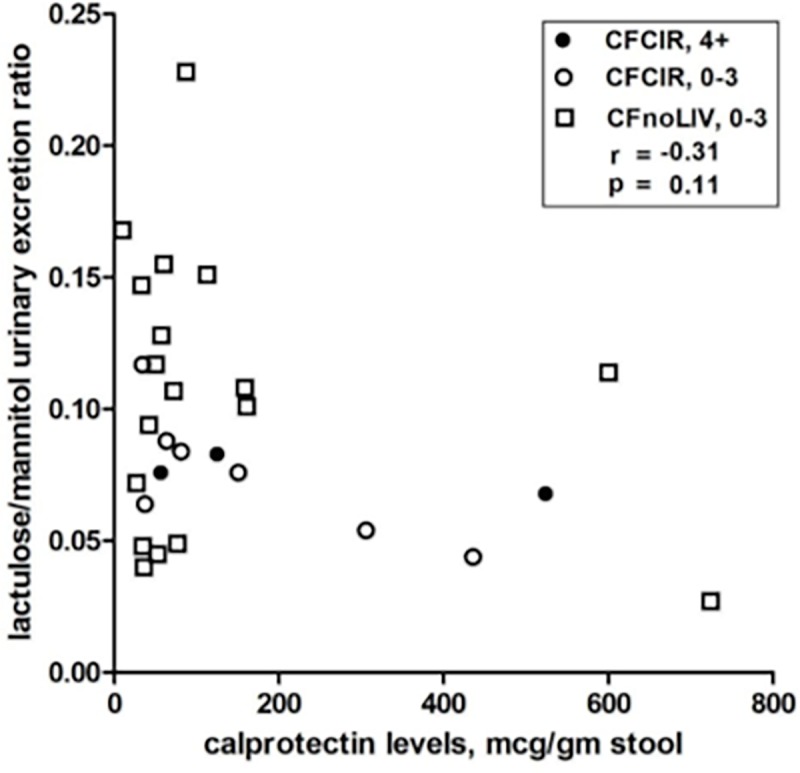
Measures of intestinal permeability and inflammation. Scatterplot comparing lactulose/mannitol urinary excretion ratio to fecal calprotectin by CFCIR (circles) or CFnoLIV (squares) stratified by significant video capsule findings (score of ≥4: solid circles) compared to mild or no findings (score of ≤3: open circles or squares). There were no CFnoLIV with a score of ≥4.

We next investigated the relationships between the key intestinal lesions by video capsule endoscopy, intestinal inflammation (calprotectin) and intestinal permeability. Fig. 2 shows the relationship between Lac/Man and calprotectin by severity of intestinal lesions and subject group. There was no correlation among Lac/Man ratio, percent lactulose or mannitol absorbed or fecal calprotectin or visual intestinal lesions or by group (CFCIR vs CFnoLIV).

**Fig 3:**
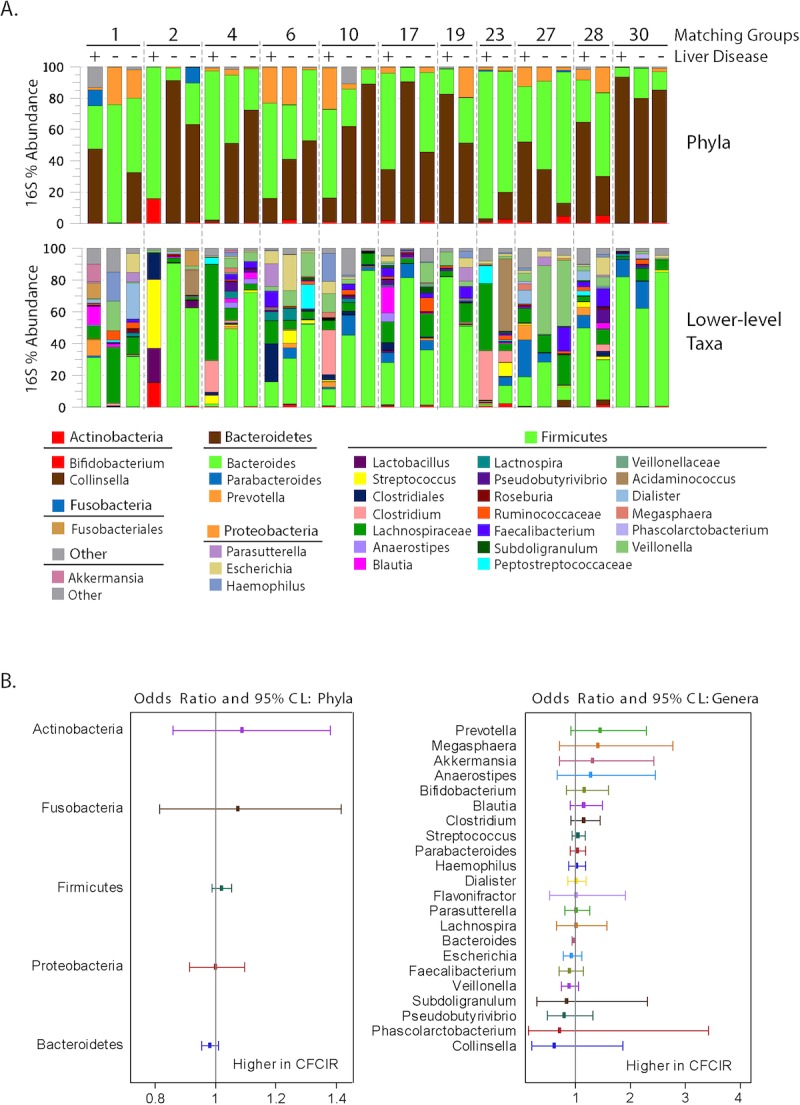
Distributions of bacterial phyla and lower-level taxa in patient fecal samples. Gut microbiomes were profiled by next-generation 16S rRNA gene sequencing. Matched samples from cases and controls are shown grouped together. For simplicity of presentation, only phyla or lower taxa (e.g., genera, families) with a minimum relative abundance of 1% are displayed. The Forest plots display the odds ratio (point) and 95% confidence intervals (whiskers) for the corresponding bacterial phyla and genera obtained from the univariate stratified logistic regressions.

**Fig 4:**
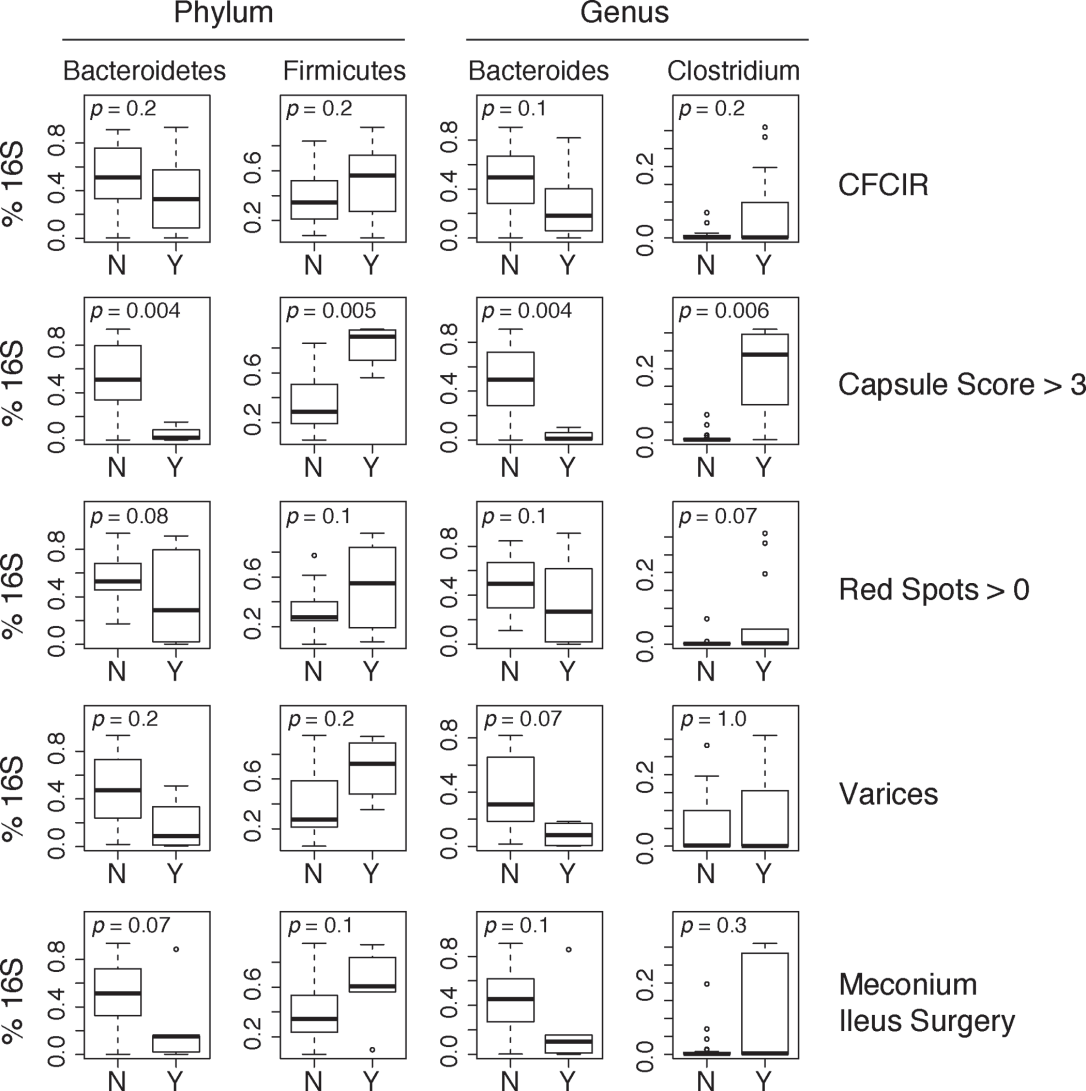
Boxplots of selected phyla and genera associated with clinical parameters.

In general, analysis of the fecal microbiomes of study participants revealed the typical bacterial profiles observed in other human studies,[56–58] with members of the phyla Firmicutes and Bacteroidetes dominating the communities (i.e., ~90% of all sequences; Table 4). Trends towards higher relative abundances (RA) of Firmicutes (51.2% vs 38.4% RA) and lower levels of Bacteroidetes (37.0% vs. 51.9% RA) were evident in CFCIR compared with CFnoLIV subjects (Table 4, Figs. 3 and 4), but the differences were not statistically significant.

At the genus level (Table 4), fecal microbiomes were dominated by *Bacteroides spp*. (40.6% RA) and diverse genera belonging to the phylum Firmicutes (43.1% RA). The genus *Bacteroides*, like the phylum Bacteroidetes, was less abundant in CFCIR than CFnoLIV (28.9% vs 47.4% RA; p = 0.10), whereas the genus *Prevotella* was more abundant in CFCIR (2.7% vs 0.4% RA; p = 0.10). Within the Firmicutes, the genus *Clostridium* was enriched in CFCIR compared with CFnoLIV (7.3% vs 0.9% RA; p = 0.19). Although not an abundant taxon, the family *Erysipelotrichaceae*, of the phylum Firmicutes, was enriched in CFCIR compared with controls (0.41% vs. 0.14% RA; p = 0.09).

**Table 4:** Stool Microbiomes of CF Subjects With and Without Liver Disease.

Taxa (Phylum, Genus)	All^a^	CFCIR^a^	CFnoLIV^a^	p-value^b^
Bacteroidetes	46.4% (30.4)	37.0% (33.2)	51.9% (28.1)	0.20
Bacteroides	40.6 (29.4)	28.9 (30)	47.4 (27.5)	0.10
Parabacteroides	3.9 (5.9)	4.6 (7.3)	3.5 (5)	0.54
Prevotella	1.2 (2.6)	2.7 (3.8)	0.4 (0.7)	0.10
Other Bacteroidetes	0.7 (1.2)	0.7 (1.5)	0.6 (1)	---
Firmicutes	43.1 (26.9)	51.2 (31.2)	38.4 (23.7)	0.21
Anaerostipes	0.5 (1.3)	0.7 (1.7)	0.4 (1)	0.44
Blautia	1.5 (3.6)	2.6 (5.7)	0.9 (1.2)	0.24
Clostridium	3.2 (8.1)	7.3 (12.5)	0.9 (1.8)	0.19
Dialister	1.4 (4.4)	1.6 (2.9)	1.3 (5.2)	0.83
Faecalibacterium	2.3 (3.9)	1.7 (3.3)	2.7 (4.2)	0.41
Lachnospira	0.9 (1.9)	0.8 (1.7)	1.0 (2)	0.94
Pseudobutyrivibrio	1.2 (1.8)	0.8 (1)	1.4 (2.2)	0.39
Streptococcus	2.8 (7.9)	5.0 (12.7)	1.5 (2.5)	0.36
Veillonella	6.9 (10.8)	3.2 (3.8)	9.0 (12.9)	0.21
Other Firmicutes	22.3 (18.1)	27.5 (22.7)	19.3 (14.8)	---
Proteobacteria	7.0 (8.8)	7.1 (9.4)	7 (8.6)	0.99
Escherichia	3.0 (4.9)	2.2 (2.9)	3.4 (5.8)	0.48
Haemophilus	1.7 (4.5)	1.9 (5.2)	1.5 (4.2)	0.76
Parasutterella	1.2 (3.2)	1.4 (4.3)	1.1 (2.6)	0.88
Other Proteobacteria	1.1 (2.1)	1.6 (2.9)	0.8 (1.4)	---
Actinobacteria	1.4 (2.9)	2.0 (4.5)	1.1 (1.5)	0.48
Fusobacteria	0.8 (2.5)	1.0 (3)	0.6 (2.3)	0.61
Verrucomicrobia	0.4 (2)	1.0 (3.3)	0.1 (0.4)	0.37
Other	0.9 (2)	0.7 (0.8)	0.9 (2.5)	---
Sequences per Subject	63748 (93454)	33160 (25906)	81457 (113116)	
Good's Coverage	99.7% (0)	99.7% (0)	99.7% (0)	
Observed Richness (Sobs)	34.5 (7.2)	35.0 (8.2)	34.2 (6.8)	
Estimated Richness (Chao1)	43.2 (8.6)	43.9 (8.4)	42.8 (8.9)	
Complexity (Shannon H)	2.48 (.88)	2.64 (.94)	2.38 (.86)	
Evenness (Shannon E)	48.2% (15.4)	51.2% (16)	46.4% (15.3)	

^a^Values are mean (std. deviation). Values for bacterial taxa are mean percent relative abundances of 16S rRNA sequence data.

^b^P-values were inferred by stratified logistic regression of relative abundance data.

**Fig 5:**
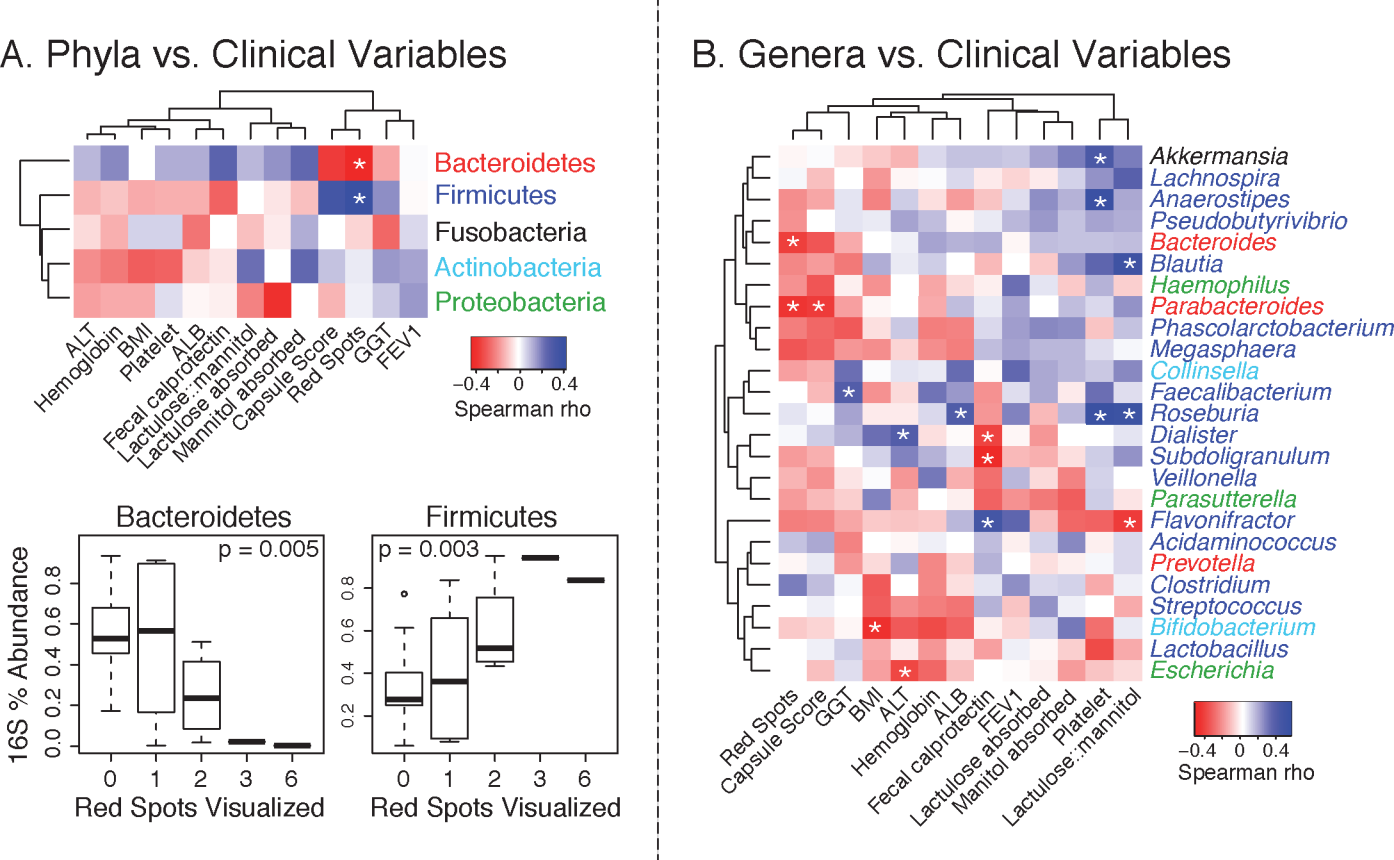
Associations of gut microbiome and clinical parameters. Associations between bacterial phyla (panel A) and lower-level taxa (e.g., genera, families; panel B) and selected clinical parameters are color-coded in the heatmaps by magnitude of Spearman’s correlation coefficient. The boxplots in panel A display the variation in relative abundances of Firmicutes and Bacteroidetes as a function of the number of intestinal red spots visualized by capsule endoscopy. Asterisks denote p-values < = 0.05 resulting from Spearman rank correlation tests.

The relative abundances of bacterial taxa (both phyla and genera) were associated with clinical variables (Fig. 5). The number of petechiae recorded by capsule endoscopy was positively correlated with Firmicutes (Spearman rho = 0.42; p = 0.023) and negatively correlated with Bacteroidetes (Spearman rho = -0.44; p = 0.016) (Fig. 5). The phylum Firmicutes was associated with more macroscopic intestinal injury (capsule score >3; p<0.01 and red spots >0 p = 0. 11) and presence of varices in the CFCIR group (p = 0.18) and having undergone meconium ileus surgery (p = 0.13) (Fig. 4). The genus *Bacteroides* was associated with less macroscopic intestinal injury (video capsule endoscopy score less than 4 (p < 0.01) and absence of red spots (p = 0.14)), absence of varices in the CFCIR group (p = 0.07) and not having meconium ileus surgery (p = 0.10). The genus *Clostridium* was associated with a higher video capsule endoscopy score greater than or equal to 4 (p = 0.01), presence of red spots (p = 0.07) and a trend with having had meconium ileus surgery (p = 0.27) (Fig. 4).

Finally, to identify clinical factors associated with the fecal microbiome community, rather than individual taxa, we performed a permutation-based multivariate analysis of variance test.[59,60] When analyzed at either the genus- or phylum-level, liver disease was significantly associated with genus-level difference in microbiome (p = 0.049) (Table 5). Capsule score, the number of red spots observed (>0 or >1), recent hospitalization for pulmonary exacerbation, prior meconium ilieus surgery, and history of varices also were significantly associated with differences in fecal microbiome, measured at both the genus- and phylum-levels (Table 5). The p-values for these variables were not appreciably affected by adjusting for liver disease (data not shown). We investigated whether the difference in inhaled antibiotics contributed to the differences observed in the relative abundance of the phyla Firmicutes and Bacteroidetes and the genera *Bacteroides* and *Clostridium* between the two groups. There was no difference in the relative abundance for any of the four taxa across antibiotic use within a group and the difference between the groups (CFCIR and CFnoLIV) remained (data not shown).

**Table 5:** Results of multivariate analysis of fecal microbiomes for single predictor variables.

	P-values^1^	
Predictor Variable	Genus	Phylum
CFCIR vs. CFnoLiv	0.049	0.20
Capsule score >3	0.0002	0.0005
Red Spots > 0	0.059	0.085
Red Spots > 1	0.0041	0.0014
Ulcers present	0.054	0.28
Gender	0.43	0.60
Pseudomonas positive	0.60	0.66
Hospitalized in the last 12 months	0.093	0.022
Allergic bronchopulmonary Aspergillosis	0.65	0.83
CF Related Diabetes present	0.20	0.33
Chronic Sinusitis present	0.24	0.34
Insulin use	0.43	0.55
Meconium ileus	0.14	0.38
Meconium ileus with surgical resection	0.022	0.094
History of varices	0.0076	0.028
Ursodeoxycholic acid current use	0.049	0.20
Azithromycin current use	0.43	0.99
Inhaled antibiotic current use	0.25	0.46
Probiotic use within 6 months	0.22	0.096

^1^Determined by permutation-based multivariate analysis of variance test of OTU count data (adonis function of vegan R package). [60] Separate analyses were performed for OTU data organized at genus-, and phylum-level. P-values less than 0.1 are highlighted in bold.

CFCIR = CF with cirrhosis, CFnoLiv = CF with no evidence of liver disease.

## Discussion

In this study we observed a higher frequency of significant macroscopic intestinal inflammatory lesions and slower small bowel transit time in CFCIR compared to age-matched CFnoLIV. Although we observed small differences in intestinal permeability between these groups, intestinal permeability was abnormal in 96% of CF subjects tested. In addition, we found significant differences in the fecal microbiome at the phylum and genus levels (results for the family level were nearly identical to the genus level) related to clinical findings of macroscopic intestinal lesions. These findings support the hypothesis that intestinal pathology and its attendant disruption of the gut microbiome may play a role in the pathogenesis of cirrhosis in CF. Slower small bowel transit time is believed to predispose to bacterial overgrowth leading to quantitative alterations and potential compositional changes in the intestinal microflora [17]. Small bowel bacterial overgrowth as measured by glucose breath test is common in CF, being present in 10–35% of individuals [61]. The increase of visible intestinal inflammatory lesions on video capsule endoscopy in our CFCIR subjects would be expected to enhance translocation of intestinal bacterial products harboring microbial associated molecular patterns (MAMPs) capable of stimulating toll like receptors on hepatic macrophages and hepatic stellate cells, triggering inflammation and fibrogenic pathways [17]. Thus, the current study provides data consistent with this potential novel mechanism for CF associated liver disease and cirrhosis.

The finding of uniformly abnormal intestinal permeability in in our subjects, all of whom were pancreatic insufficient is consistent with prior reports in subjects with CF and pancreatic insufficiency [26]. The underyling pathophysiology for this observation is unclear, but may involve injury to the small bowel mucosa from a more acidic pH due to the lack of endogenous buffering capacity in pancreatic insufficiency, impaired functional integrity related to CFTR dysfunction or injury from exogenous pancreatic enzymes [26]. Thus, the small intestine in CF is primed to absorb microbial products that could potentially trigger hepatic inflammatory signaling and fibrogenesis pathways. It is hypothesized that either differences in the severity of intestinal inflammation, small intestinal bacterial overgrowth or microflora composition in conjunction with this increased permeability may combine as a second factor in the pathogenesis of CF liver disease. Indeed recent work has demonstrated that in the CF mouse model, intestinal inflammation can result in hepatic injury [18,19].

Intestinal inflammation, as determined by fecal calprotectin, was common in this unselected group of subjects with CF; 45% of CFCIR and 26% of CFnoLIV had an elevated fecal calprotectin. Interestingly, there was no correlation between fecal calprotectin and either intestinal permeability measured by Lac/Man or macroscopic intestinal lesions by video capsule endoscopy (Fig. 2). It should be noted that swallowed inflammatory cells from airway secretions could also have accounted for the fecal calprotectin.

The fecal microbiotas of the CFCIR group trended towards relatively higher abundances of Firmicutes, and concomitantly lower abundances of Bacteroidetes, compared with the control group. Similar shifts in the Firmicutes/Bacteroidetes ratio have been observed in some, but not all, studies of obesity [62,63] and fatty liver disease [64,65]. For instance, Mouzaki et al reported decreased Bacteroidetes in patients with biopsy-proven nonalcoholic steatohepatitis [66]. Similarly, Raman et al reported that several genera of Firmicutes were enriched in obese individuals with nonalcoholic fatty liver disease [67]. In contrast, Zhu et al found higher Bacteroidetes and lower Firmicutes in both obese and NASH individuals relative to controls, highlighting the potential indiosyncrasies of studying the human microbiome in the context of complex, multi-factorial diseases [68]. At the genus level, increases in *Clostridium* have been reported in cirrhotic patients compared to healthy controls [69] and were found to be increased in our subjects with more macroscopic intestinal inflammation (Fig. 4). Of note, expansion of this bacterial group has been associated with obesity and fatty liver disease in humans and animal models [70–73]. For instance, Spencer et al. reported that the abundance of *Erysipelotrichia* was a positive predictor of liver fat accumulation in humans experimentally subjected to a low choline diet [71]. Analysis of *Erysipelotrichia* genomes indicates that the evolutionary process of genome reduction has resulted in the loss of fatty acid biosynthesis genes [74,75]. Because shifts in the relative abundance of these organisms in the gut may simply reflect growth rates that are limited by luminal fatty acid availability, further experimentation is required to establish whether the *Erysipelotrichias* contribute to liver disease.

The differences in the microbiome in subjects with and without varices suggest that this may be a potential therapeutic target in CF liver disease. Although matching cases and controls by history of pseudomonas infection at least partially controlled for antibiotic-mediated effects on the microbiome, we cannot rule out the possibility that the altered microbiomes arose in response to other medical treatments specific for CF patients with liver disease. There were no differences between recent hospitalizations or use of chronic azithromycin use between CFCIR and CFnoLIV. While there was a difference in the use of inhaled antibiotics, we did not find an effect on the distribution of phyla or genera between CFCIR or CFnoLIV.

In our study, we could not determine if there was a direct causal link between the intestinal lesions, fecal microbiome or changes in the small bowel transit time and the development of cirrhosis in CF. [76] For example, increased intestinal permeability may be secondary to portal hypertension, vascular congestion of the small intestine and molecular alterations in gap function proteins that have been described during cirrhosis. Nevertheless, the substantial evidence for involvement of the gut-liver axis in animal models of cholestasis, steatohepatitis and cirrhosis and the supportive evidence from human studies [17] provide the justification to further pursue this possibility in CF liver disease. The alterations in the fecal microbiome of subjects in the current study provide further evidence for this hypothesis.

The limitations of this study include a small sample size. This was designed as a pilot study to determine if further investigation of this hypothesis has merit in CF. We determined the small bowel transit time by capsule endoscopy with a standard preparation, but as a consequence, the subjects received a short course of Miralax and a small dose of erythromycin both of which may affect small bowel motility [77–79]. However all subjects received the same preparation balancing for the potential medication effects on small bowel transit. In addition we could not determine if the lesions in the intestine, alterations in the fecal microbiome and abnormalities of motility and inflammation are primary to the development of cirrhosis or secondary effects. A prospective assessment of these factors should be considered as part of longitudinal studies of the development of cirrhosis in CF. Furthermore, reliance on fecal sampling limits the ability to detect microbiome changes in proximal segments of the GI tract, for instance the small intestine, which might be more directly involved in hepatic disease.

In conclusion, this study provides the first evidence of a link between alterations in the CF intestine, fecal microbiome and development of advanced CF liver disease. The evidence supporting the gut-liver axis model in related liver diseases is gaining merit; our study suggests that CF is another potential disorder in which interactions between disturbances in intestinal integrity and hepatic inflammatory and fibrogenesis pathways may be active. This knowledge may offer a new window of early therapeutic interventions to mitigate the systemic effects of these alterations.
